# Supplementary material for: Molecular Characterization, Expression Pattern, DNA Methylation and Gene Disruption of Figla in Blotched Snakehead (Channa maculata)
Source: Animals (Basel). 2024 Feb 1;14(3):491. doi: 10.3390/ani14030491 (PMC10854511; doi:10.3390/ani14030491)
Supplement: Supplementary file 1 [file animals-14-00491-s001.zip › animals-2836757-supplementary.pdf]

Figure S1

(a)

```
   1 ggactgttaggggttcagtgtcttgcccagggacacttcgacctatagccagggccgggatcgaaccactcaccctatggtccgtggacgactgcc
  97 tttaccaactgagcgaatacgtttggcaatatagtgtagtttttgaaagttactttatagtgttcgaaatatctgaaactgtttaaaatcttta
 193 aatccaaatgtgccaccctttttctagatccgcccctgacgtcgtgatggacacttggacagcctcacgtcaggtagccgtgggcagagggctaatt
 289 aaacgtggagcacaggtgtgatgggcccagctcgccctcgtgcagcctctgccctttgtgtttccatgcggactgagtggaaaacacactgctaga
   1                                                    M  K  V  P  E  E  E  L  M  S  D  I  L  M  R  L  T
 385 aagtaacagtctgtcaggttgggtatatgttagcattccgtgttgaaATGAAGGTGCCAGAGGAGGAATTAATGAGTGACATTTTGATGCGTTTAA
  18  G  E  S  A  L  P  V  Y  S  N  I  E  K  F  R  R  T  K  D  G  L  Y  F  V  A  E  E  F  S  E  T  V
 481 CGGGCGAGTCCGCCCTCCCCGTATACAGCAACATTGAGAAATTCAGACGGACTAAGGACGGCTTGTACTTCGTAGCCGAAGAATTCAGTGAAACTG
  50  K  K  R  E  L  V  N  A  K  E  R  L  R  I  R  N  L  N  T  M  F  S  R  L  K  R  M  V  P  L  M  R
 577 TCAAAAAGAGAGAATTGGTCAACGCCAAGGAACGACTGAGAATCCGGAACTTGAACACCATGTTTTCCCGCTTAAAGCGCATGGTGCCACTTATGC
  82  P  D  R  K  P  S  K  V  D  T  L  K  A  A  T  E  Y  I  R  L  L  V  A  V  L  Q  D  A  D  S  D  D
 673 GACCAGACCGCAAACCCAGTAAAGTCGACACACTCAAAGCTGCAACAGAATACATCCGATTGCTTGTTGCAGTTTTGCAGGATGCTGACAGTGATG
 114  G  S  G  T  D  F  L  K  N  A  I  T  Y  G  Q  T  D  G  L  G  N  D  L  W  R  V  D  D  L  L  N  M
 769 ATGGCAGTGGGACTGATTTCCTAAAGAATGCAATCACTTATGGTCAGACTGATGGCTTGGGCAATGACCTATGGAGAGTGGATGATCTGCTAAACA
 146  S  D  E  H  M  E  D  G  F  A  M  P  P  E  P  V  A  E  D  G  D  M  S  R  L  V  L  Q  H  C  V  M
 865 TGTCAGATGAGCATATGGAAGATGGATTTGCAATGCCCCCAGAACCGGTAGCGGAGGATGGAGATATGAGCAGGCTGGTGTTGCAACACTGTGTGA
 178  P  A  Y  Q  F  I  I  Q  L  A  P  D  Q  S  S  M  S  Q  P  C  *
 961 TGCCTGCGTACCAATTCATCATCCAACTAGCACCTGATCAGTCTTCGATGTCGCAACCCTGCTGAgtggtgtttagagcagatggcttctacaagt
1057 acaagcttctacaaacgggcctgttgctttgacagaagaatcttgaagaaaatgttttaattttactttgaaattcaaatttgtttgtgtttctc
1153 taaaatataatttcacagctgtatctttggcagccctcaaataaatttacaggacagtgtcatttgtttttctattaagtgtttgttttcaagaa
1249 aaataaaacagattttatatattcagaaaaaaaaaaaaaaaaaaaaaaaaaaaa
```

(b)

```
-1003 ctttgttgctactaagttgctgtgtcacttattatgtattgtctaggatcaaaaaattgatttatcttcagatttatctg
-923  tacattgactttttttgcatcaaaaacatcagattgttgggtatttgactgatgcatcgatcaagattgatggatagttag
-843  acccctaagagccggaaggggagacaccaaccaaatctgattgagtgtaaaggggttggtacctgttgtagtactttcag
                                                      Sp1
-763  caaaagaaaaattgaccaaatctgaagctgaaataaatgaccataattatagggaaaataaataatactattttgtacat
                                                                  SOX3
-683  ttaagatgattttacacattttgtagcagttgcaaccaggcacattatcccttcaagggatggattttagctttctccaa
-603  ctgtttatgattatgtgtttgactactttcagtaaaaaaaaaaaagaaaaaaacatgttcggtatttggcatttctagaat
-523  aacaccttgaaacacactattattatttcacagtttatttatatctgaagctgtatttttttttttttttttttttttt
                    SRY
-443  ttttctttcggcttatcccgtgagttcagggtcgccacagcggatcattgtccgcatgttgatttggcacagttttacg
-363  ccggatgctcttcctgacgcaaccctccccaatttctaccgggcagctggggaggggaatggactgttaggggttcagtg
                                  Sp1                 Sp1
-283  tcttgcccagggacacttcgacctatagccagggccgggatcgaaccactcaccctatggtccgtggacgactgccttta
                  Esr2
-203  ccaactgagcgaatacgtttggcaatatagtgtagtttttgaaagttactttatagtgttcgaaatatctgaaactgtt
-123  taaaatctttaaatccaaatgtgccaccctttttctagatccgcccctgacgtcgtgatggacacttggacagcctcacg
                                                  Sp1
-43   tcaggtagccgtgggcagagggctaattaaacgtggagcacaggtgtgatgggcccagctcgccctcgtgcagcctctgc
                                                   +1
+37   cctttgtgtttccatgcggactgagtggaaaacacactgctagaaagtaacagtctgtcaggttgggtatatgttagcat
         SOX3
+117  tccgtgttgaaATGAAGGTGCCAGAGGAGGAATTAATGAGTGACATTTTGATGCGTTTAACGGCGAGTCCGCCCTCCCC
                                                                            Esr2   Sp1
+197  GTATACAGCAACATTGAGAAATTCAGACGGACTAAGGACGGCTTGTACTTCGTAGCCGAAGAATTCAGTGAAACTGTCAA
+277  AAAGAGAGAATTGGTCAACGCCAAGGAACGACTGAGAgtgagtttttgtaataacactactccattattggtgtgtttga
+357  cgggatgatggctaagataagtcaggttccgtcagaacttactcacttaggtccaacattaacctcagctaacaagtagt
+437  acgttagcaacctaaatatgttatcgcgcatactgtgttttgtgtgtgcgcatgtatctgtcaatattccacagatt
```

Figure S2

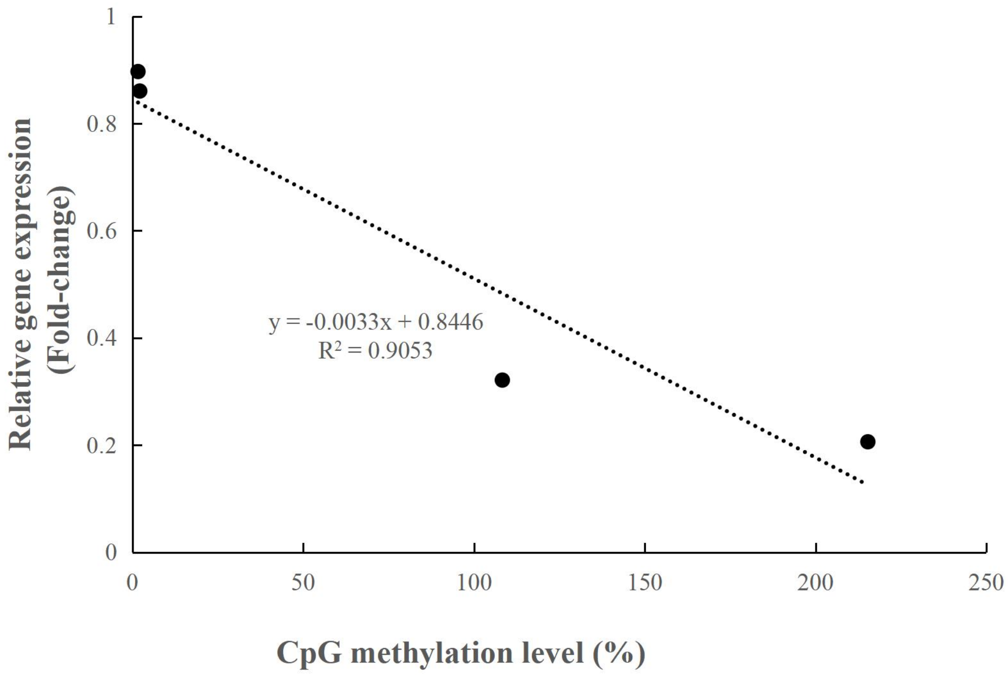

1
0.8
0.6
0.4
0.2
0
0
50
100
150
200
250
Relative gene expression (Fold-change)
y = -0.0033x + 0.8446
R² = 0.9053
CpG methylation level (%)
